# Lectures on the Diseases of Infancy and Childhood

**Published:** 1850-01

**Authors:** 


### ARTICLE II.

*Lectures on the Diseases of Infancy and Childhood.*---By Chas. West, M.D., Fellow of the Royal College of Physicians. Page 452: octavo. Philadelphia: Lea & Blanchard. 1850. (From the Publishers, by S. C. Griggs & Co., Chicago.)

This work, which recently came out in the Medical News and Library, in numbers, is now before us entire, and is a highly valuable treatise.

Although we should be in favor of an International Copy Right Law by Congress, to prevent the appropriation of the labors of others to our own use without paying them any thing in return, and to give our own authors an opportunity to enter the field in competition ; sill, while there is no such restriction, and books will be republished here without paying any thing for the Copy-Right, as we is the case with foreign works, we are glad that such good books as this are selected, for our publishers cannot be accused of furnishing an inferior article, if it does come cheap.

The operation of this system of republication upon our native talent for writing, both in book and periodical publication, is most destructive to our national medical literature. As it now is, the American author is not only under obligations to write for nothing, to compete with foreign productions that come on those terms, but he must produce a better work than can be found in Europe, or his work will receive no attention. In this, the advantages, it is ture, are on our side, as works written here, might, *a priori*, be expected to suit the wants of the physician ot this country on account of the peculiarities and modifying influences on diseases that are found in the climate, constitution and habits of the people, and the existence of new forms of disease in this country.---But notwithstanding these differences, which we think all of our countrymen will asert to exist, there are many who will buy the foreign work, of the same comparative merit, in preference to the native producton.

Then let us have a Copy Right Law, that will foster a national medical literature in America. E.

